# Hierarchical decomposition of dynamically evolving regulatory networks

**DOI:** 10.1186/s12859-015-0529-9

**Published:** 2015-05-15

**Authors:** Ahmet Ay, Dihong Gong, Tamer Kahveci

**Affiliations:** 1 Departments of Biology and Mathematics, Colgate University, Hamilton, 13346 NY USA; 2 Department of Computer and Information Science and Engineering, University of Florida, Gainesville, 32611 FL USA

**Keywords:** Hierarchy, Gene regulatory networks, Network dynamics

## Abstract

**Background:**

Gene regulatory networks describe the interplay between genes and their products. These networks control almost every biological activity in the cell through interactions. The hierarchy of genes in these networks as defined by their interactions gives important insights into how these functions are governed. Accurately determining the hierarchy of genes is however a computationally difficult problem. This problem is further complicated by the fact that an intrinsic characteristic of regulatory networks is that the wiring of interactions can change over time. Determining how the hierarchy in the gene regulatory networks changes with dynamically evolving network topology remains to be an unsolved challenge.

**Results:**

In this study, we develop a new method, named *D-HIDEN* (Dynamic-HIerarchical DEcomposition of Networks) to find the hierarchy of the genes in dynamically evolving gene regulatory network topologies. Unlike earlier methods, which recompute the hierarchy from scratch when the network topology changes, our method adapts the hierarchy based on the wiring of the interactions only for the nodes which have the potential to move in the hierarchy.

**Conclusions:**

We compare D-HIDEN to five currently available hierarchical decomposition methods on synthetic and real gene regulatory networks. Our experiments demonstrate that D-HIDEN significantly outperforms existing methods in running time, accuracy, or both. Furthermore, our method is robust against dynamic changes in hierarchy. Our experiments on human gene regulatory networks suggest that our method may be used to reconstruct hierarchy in gene regulatory networks.

## Background

Regulatory interactions between genes in a cell are often represented as a network, also called gene regulatory network. These networks are often modeled as directed graphs where nodes represent the genes or their products, and directed edges between these nodes represent the regulation of a gene by another one. Gene regulatory networks govern almost every biological activity in the cell [1-8]. Due to their central role in the development of organisms and human diseases, the analysis of gene regulatory networks holds the key for understanding how biological processes are regulated. Subtle changes, such as an increase or decrease of the abundance of regulatory proteins or aberrations in regulatory interactions between these proteins, can have profound effects in many biological processes including human diseases such as cancer.

Biological networks have been characterized by a variety of graph theoretic measures such as degree distribution and clustering coefficient [9]. Analysis of the gene regulatory networks has shown that these networks share common characteristics such as the scale-free degree distribution and hierarchical ordering of the genes [10-20].

**Figure 1:**
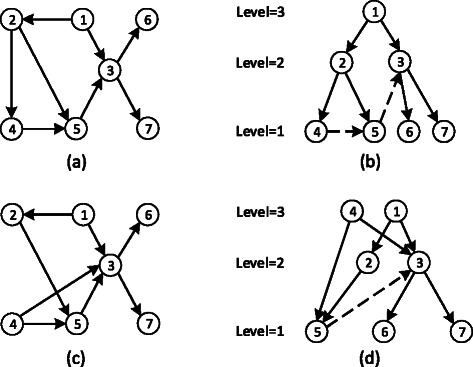
Hierarchical decomposition of gene regulatory networks.**(a)** A hypothetical regulatory network with seven nodes, labeled with ***1,2,…,7***. **(b)** The same network after nodes are assigned to three hierarchy levels with optimal hierarchical assignment. Dashed arrows indicate conflicting edges. There are two conflicting edges. Thus the penalty of this decomposition is 2. **(c)** The network obtained by mutating the network in **(a)** after removing edge (2,4) and inserting edge (4,3). **(d)** The optimal hierarchical decomposition of the network in **(c)** into three hierarchy levels.

Hierarchical decomposition reveals one of the most fundamental characteristic of gene regulatory networks. It explains the flow of information (i.e., regulation in this case) from the genes at higher levels (i.e., master regulators) to those at lower levels [11,13]. More specifically, hierarchical decomposition maps the nodes in the underlying network to one of the given hierarchy levels, typically denoted with integers 1, 2, …, *M*. Here, *M* denotes the highest possible hierarchy level. Figure 1 illustrates this concept. Figure 1(a) shows a hypothetical network. Figure 1(b) illustrates the same network after the nodes are assigned to three hierarchy levels.

Correct assignment of the hierarchy levels to the genes in the regulatory networks is essential for comprehensive analysis of the gene regulatory networks, particularly for understanding the impact of external perturbations and diseases on the transcription and abundance of genes and gene products. While the experimental approaches can be used to identify the genes and their interactions, finding the hierarchy between these genes remains to be a computational task.

Some of the key approaches for finding the hierarchy of genes in regulatory networks computationally include BFS [11], Vertex-Sort [12], HINO [13], HIDEN, and Divide and Conquer HIDEN (DC-HIDEN) [14]. The BFS method uses breadth-first search technique to assign hierarchies to genes in a network [11]. Although this method is applicable to any gene regulatory network, it fails to correctly assign hierarchy levels for networks that contain cycles. Vertex-Sort uses a topological sort algorithm to assign hierarchies to genes in the given network [12]. This method does not assign a single hierarchy level for each gene; it instead assigns a range of possible levels for genes. HINO improves the BFS method by introducing two correction steps, called “downgrade” and “upgrade”. Downgrade step places each node at the lowest level where that vertex and its regulating nodes are assigned to. Upgrade step assigns nodes to the next higher level if they regulate other nodes which are located at the same level and no other node regulates them. Despite this improvement, its accuracy remains very low. HIDEN [14] formulates the problem using integer linear programming. In comparison to BFS, Vertex-Sort and HINO, HIDEN produces more accurate hierarchy assignments. However, this comes at the price of significantly increased computational cost. As a result, HIDEN does not scale to large gene regulatory networks. To remedy this shortcoming, the same study, proposes a divide and conquer strategy, named DC-HIDEN. This strategy computes a set of local solutions for randomly selected subnetworks of the given network and then combines them. As a result of this localization, this method scales to large networks, but its accuracy levels are lower in comparison to that of HIDEN. In summary, all of these aforementioned approaches are either grossly inaccurate or take enormous amount of time as the network size grows. As we explain next, such enormous cost leads to another challenge that makes these methods impractical.

An inherent characteristic of gene regulatory networks is that their topologies are dynamic. New interactions can appear or existing interactions can become infeasible over a period of time [21]. For example, many genes are involved in regulatory networks that control differentiation in different cell types during early development. The interactions between these genes vary throughout development and in different cell types. One reaction that exists at one time point may be lost at a later time point, or a reaction that exists in one cell type may be missing in another cell type. These dynamic changes in the regulatory interactions reflect the changes in the needs of the system and are key for correct development of the organism. After the organism develops into its adult form, cell differentiation is minimal and most of the regulatory interactions that are involved in these regulatory pathways are lost. However, the dynamic rewiring of the gene regulatory networks for other cellular functions is still needed to efficiently respond to environmental changes [22-24]. Clearly, such topological changes in the regulatory networks can alter the position of a subset of genes in the hierarchical decomposition, yet we do not know which genes will change or how they will change their hierarchies [15-20]. Determining such topological changes is key for understanding how gene regulatory networks function, how the development works for different organisms and finding cures for diseases such as cancer.

To the best of our knowledge, all the methods in the literature targeted at computing the hierarchical decomposition of biological networks ignore the dynamic nature of the network topologies. As a result, they cannot handle dynamic changes in the gene regulatory networks. An obvious way to apply these methods on dynamic network topologies is to recompute the hierarchical decomposition from scratch each time the network topology changes, even when the change is miniscule. This is, however, not practical as finding the hierarchical decomposition is a computationally expensive task. New methods that can quickly update the hierarchical decomposition from the existing hierarchy when network topology is updated slightly are needed.

**Our contributions** In this paper, we develop a new method, namely *Dynamic Hierarchical Decomposition of Regulatory Networks (D-HIDEN)* which finds the hierarchy in gene regulatory networks. Unlike existing methods, D-HIDEN can handle networks whose topologies change dynamically. The idea behind D-HIDEN is that small changes in the topology of a network alters the hierarchies of only a small number of nodes of that networks. When the network topology changes, instead of recomputing the hierarchy from scratch for the entire network, D-HIDEN computes the hierarchy levels of a small part of the underlying network that is most likely to move in the hierarchy. We formulate this problem as an integer linear programming problem. The challenge here is to predict which subnetwork yields the variables in the resulting mixed integer linear programming. D-HIDEN tackles this problem by learning a function that describes the probability that a given gene in the network will move in the hierarchy, based on a given set of alterations in the network topology (i.e., insertions and deletion of interactions). Our extensive experimental results on both synthetic and real networks demonstrate that D-HIDEN achieves much better performance (accuracy and running time) than five currently available methods. Applications of our method to the human gene regulatory networks could successfully reconstruct the hierarchy in these networks. Our analysis on the human gene regulatory networks evolving due to cell differentiation reveals changes in the hierarchy of the regulatory roles of genes.

The implementation of the methods we developed in this paper and the datasets we used in our experiments are available at http://bioinformatics.cise.ufl.edu/dhiden.

The rest of the paper is organized as follows. In the Methods section, we formally define the problem and describe the D-HIDEN algorithm. In the Results section, we present the results of our method. In the Discussion and Conclusion section we summarize our findings.

## Methods

In this section, we first present the key terms that are essential to describe our method. We then explain our method in detail.

### Terms and definitions

A gene regulatory network describes the regulatory interactions between genes and their products. Mathematically, we model a gene regulatory network using a directed graph denoted with *G*=(*V*,*E*). In this notation, *V* denotes the set of nodes where each node corresponds to a unique gene. *E*⊆*V*×*V* denotes the set of directed edges where each edge corresponds to an interaction. In the rest of this paper, we will use the term graph to denote directed network unless otherwise stated explicitly. We start by defining the inversion operation on graphs and how we use it to enrich a graph.

#### **Definition1** (Inversion of a graph).

Given a graph *G*=(*V*,*E*), we say that graph *G* ^*i*^=(*V*,*E* ^*i*^) is the *inverse* of *G* if *E* ^*i*^ is obtained by reversing the directions of all the edges in *E*. Formally, this happens when both of the following two conditions hold: (i) ∀(*u*,*v*)∈*E*, we have (*v*,*u*)∈*E* ^*i*^, and (ii) ∀(*u*,*v*)∈*E* ^*i*^, we have (*v*,*u*)∈*E*.

#### **Definition2** (Direction enriched graph).

Consider a graph *G*=(*V*,*E*) and its inverse *G* ^*i*^=(*V*,*E* ^*i*^). We say that graph *G* ^*e*^=(*V*,*E* ^*e*^) is *direction enriched* graph of *G* if *E* ^*e*^=*E*∪*E* ^*i*^.

We say that a graph *G*=(*V*,*E*) is *connected* if its direction enriched graph contains at least one path from all the nodes of *V* to all the other nodes of *V*. In the rest of this paper, unless otherwise specified, we assume *G* to be directed and connected. It is worth noting that our method does not rely on this assumption. We only make this assumption to simplify the method description. We defer the discussion on disconnected graphs to the end of this section.

#### **Definition3** (Hierarchical decomposition of a graph).

Consider a graph *G*=(*V*,*E*), and a positive integer *M* denoting the highest possible hierarchy level. The *hierarchical decomposition* of *G* is a function *H*:*V*→{1,…,*M*}.

Verbally, the hierarchical decomposition assigns a level, which is described by an integer, to the nodes of the graph. In this hierarchy, larger numbers indicate a higher level in the hierarchy, and thus, *M* is the highest level of hierarchy. The Figure 1(b) shows an example of hierarchical decomposition where *M*=3.

The hierarchical decomposition of a graph describes the relative role of the genes as the regulators or the regulated ones in that graph. Ideally, if a gene denoted by node *u* regulates another gene denoted by node *v* (i.e., there is a directed edge from *u* to *v* in the corresponding graph), then we would like to place node *u* at a higher level in the hierarchy than node *v*. The following two definitions capture this.

#### **Definition4** (Conflicting edge).

Consider a graph *G*=(*V*,*E*), and its hierarchical decomposition, *H*:*V*→{1,…,*M*}. Consider an edge (*u*,*v*)∈*E*. We say that the edge (*u*,*v*) is *conflicting* if *H*(*u*)≤*H*(*v*).

For instance, in Figure 1(b), the edge (4,5) is a conflicting edge because *H*(4)=*H*(5). Similarly, the edge (5,3) is also conflicting as *H*(5)<*H*(3).

#### **Definition5** (Penalty of hierarchical decomposition).

Consider a graph *G*=(*V*,*E*), and its hierarchical decomposition, *H*:*V*→{1,…,*M*}. For all (*u*,*v*)∈*E*, consider the indicator variable *ε*_*uv*_ defined as

$$\epsilon_{uv}=\left\{ \begin{array}{c l} 1 & H(u) \leq H(v)\\ 0 & otherwise \end{array}\right. $$

The *penalty* of *H* on the graph *G* is the number of conflicting edges in *G* based on the hierarchy function *H*. Formally, it is computed as:

$$P_{G}(H)=\sum_{(u,v) \in E}\epsilon_{uv} $$

For instance, in Figure 1(b), penalty of the given hierarchy is two since there are two conflicting edges. We say that a hierarchical decomposition of a given graph into *M* hierarchical levels is *optimal* if it yields the least penalty among all possible hierarchical decompositions.

#### **Definition6** (Dynamically evolving graphs).

Assume that we are provided with a positive integer *τ*. Also assume that we are given sequences of graphs **G**=(*G*_1_,…,*G*_*K*_), where ∀*i*(1≤*i*≤*K*), *G*_*i*_=(*V*_*i*_,*E*_*i*_). We say that **G** is a sequence of *dynamically evolving graphs* with respect to *τ* if both of the following two criteria are satisfied for consecutive graphs *G*_*i*−1_ and *G*_*i*_ in **G**, ∀*i*(1<*i*≤*K*),

1. *V*_*i*−1_=*V*_*i*_,
2. |*E*_*i*−1_−*E*_*i*_|+|*E*_*i*_−*E*_*i*−1_|≤*τ*.

In Definition 6 above, the first condition enforces that the set of nodes should be preserved (we will relax this constraint later in this section). The second condition requires that only a limited amount of edges can be altered (i.e., inserted or removed) from one graph to obtain the next graph in the sequence.

When the threshold *τ* in Definition 6 is small, instead of considering the *K* graphs in the dynamically evolving sequence **G** as *K* independent graphs, we view them as single graph whose topology is gradually changing from *G*_1_ to *G*_2_, then to *G*_3_, and so on until we reach *G*_*K*_.

Notice that as the graph topology evolves as provided in the sequence **G**, the hierarchy level assigned to the nodes of the graph by the optimal hierarchical decomposition can also change. This is because as new edges are inserted or existing ones are removed, the role of the gene (i.e., node) incident to those edges as the regulator of other genes can change. For instance, in Figure 1(a), if we remove edge (2,4) and insert edge (4,3), we obtain the graph in Figure 1(c). Figure 1(d) presents the optimal hierarchical decomposition of the resulting graph. Notice that the hierarchical decomposition in Figure 1(d) differs from that in Figure 1(b). Following from this observation, we define two sets of nodes based on the graphs corresponding to two consecutive graphs of a given dynamically evolving graphs next.

#### **Definition7** (Dynamic and complementary nodes).

Consider two consecutive graphs *G*_*i*−1_ and *G*_*i*_ in a sequence of dynamically evolving graphs. Let us denote their optimal hierarchical decomposition with *H*_*i*−1_ and *H*_*i*_ respectively.

We define the subset of nodes whose hierarchy levels change as *G*_*i*−1_ evolves to *G*_*i*_ as the *dynamic node set* and denote it with *D*_*i*_. Formally

$$D_{i}=\left\{v|v \in V_{i}, H_{i-1}(v) \neq H_{i}(v)\right\} $$

We define the nodes which are not in *D*_*i*_ but have direct connections to at least one of the nodes in *D*_*i*_ as the *complementary set* and denote it with *C*_*i*_. Formally

$$C_{i}=\left\{v|v \in V_{i} \backslash D_{i}, \exists u \in D_{i} \: s.t. \: (u,v) \in E_{i} \vee (v,u) \in E_{i}\right\} $$

We are now ready to define the problem considered in this paper formally.

**Formal problem definition** Given a sequence of dynamically evolving graphs **G**=(*G*_1_,*G*_2_,...,*G*_*K*_), our aim is to find an optimal hierarchy assignment for all graphs in **G**. In other words, for each graph *G*_*i*_ in **G**, we would like to find *H*_*i*_ such that

$$H_{i}={argmin}_{H}\left\{P_{G_{i}}(H)\right\} $$

The naive solution to the problem above is to compute the hierarchical decomposition for each graph *G*_*i*_ in **G** independently using existing algorithms, such as HIDEN [14]. This is however not practical as the size of the graph (number of nodes and edges) and the number of graphs in **G** increases, the cost of finding the optimal hierarchical decomposition grows rapidly.

### Our algorithm: Dynamic-HIDEN

**Figure 2:**
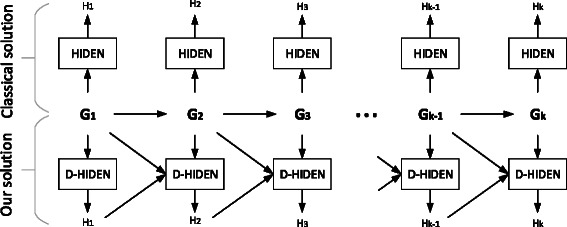
Illustration of the key difference between D-HIDEN and the classical hierarchical decomposition algorithms, such as HIDEN. The HIDEN method in the figure can be replaced by any existing hierarchical decomposition method without losing generality. HIDEN computes the hierarchy of all the networks *G*_*i*_ independently. On the other hand, D-HIDEN updates the hierarchy of each network *G*_*i*_ based on the topological difference between *G*_*i*_ and *G*_*i*−1_, and the existing hierarchy *H*_*i*−1_ of *G*_*i*−1_.

Here, we describe our method, named *Dynamic-HIDEN* (D-HIDEN), which computes the hierarchial decompositions of the graphs in a given sequence of dynamically evolving graphs **G**=(*G*_1_,…,*G*_*K*_). The central idea behind our method is our conjecture that if the topology of the underlying graph does not change significantly from one graph in the sequence to the next, then their hierarchical decomposition also does not change dramatically. Following from this conjecture, our method computes the hierarchical decomposition of the first graph *G*_1_ similar to the HIDEN method. To compute the hierarchical decomposition for the remaining graphs in **G**, say *G*_*i*_ with 1<*i*≤*K*, it exploits two kinds of information: the part of the topology that is different between consecutive graphs *G*_*i*−1_ and *G*_*i*_ into consideration as well as the hierarchical decomposition of *G*_*i*−1_ denoted with *H*_*i*−1_. Figure 2 illustrates this idea. We elaborate on our algorithm next.

#### Integer linear programming formulation of hierarchical decomposition

D-HIDEN computes the hierarchical decomposition of each graph in a sequence of dynamically evolving graphs **G** using Integer Linear Programming (ILP). Unlike, existing methods that also use ILP, it creates variables only for a small set of nodes and edges. As we will explain later in detail, these variables correspond to the nodes that will change their position in the hierarchical decomposition with a high probability. We present the ILP formulation next by focusing on the *i*th graph in the sequence denoted by graph *G*_*i*_=(*V*_*i*_,*E*_*i*_).

Suppose that we are given the hierarchy assignment *H*_*i*−1_ for the graph *G*_*i*−1_, and our aim is to compute a new hierarchy assignment *H*_*i*_ for the graph *G*_*i*_. We start by constructing an induced subgraph $S_{i}=({V_{i}^{s}},{E_{i}^{s}})$ using the dynamic and complementary node sets *D*_*i*_ and *C*_*i*_ as follows:

$$\begin{aligned} {V_{i}^{s}}&=D_{i} \cup C_{i}\\ {E_{i}^{s}}&=\left\{(u,v)|u \in {V_{i}^{s}}, v \in {V_{i}^{s}}, (u,v) \in E_{i}\right\} \end{aligned} $$

**Figure 3:**
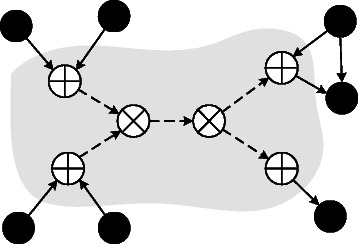
Illustration of the relationship between the dynamic and the complementary node sets for graph ***G***_***i***_. The nodes shown with $\bigotimes $ denote the dynamic node set *D*_*i*_. The set of nodes connected to at least one node in *D*_*i*_ (shown with $\bigoplus $) constitute the complementary node set *C*_*i*_. Dashed edges are in the edges in the set ${E_{i}^{s}}$.

Figure 3 illustrates construction of subgraph *S*_*i*_. Notice that knowing the sets ${V_{i}^{s}}$ and ${E_{i}^{s}}$ requires knowing the hierarchical decomposition *H*_*i*_ of *G*_*i*_, which is actually the problem we are trying to solve here. We discuss how we predict the sets *D*_*i*_ and *C*_*i*_ to construct the graph *S*_*i*_ prior to computing *H*_*i*_ later in this section. We continue explaining the ILP formulation.

We keep the hierarchy assignments of all the nodes in *V*_*i*_−*D*_*i*_ the same as the previous graph’s hierarchy assignment *H*_*i*−1_ (i.e., ∀*v*∈*V*_*i*_−*D*_*i*_,*H*_*i*_(*v*)=*H*_*i*−1_(*v*)). We only compute the hierarchy assignments for the nodes in *D*_*i*_ by solving the following optimization problem.

$$\begin{aligned} Minimize: \qquad\qquad\qquad &{\sum\nolimits}_{(u,v) \in\, {E_{i}^{s}}}\epsilon_{uv}\\ Subject ~~ to: \qquad\qquad\qquad t_{u} &- t_{v} + \epsilon_{uv}\, M>0\\ \epsilon_{uv} \in &\{0,1\} \: \forall (u,v) \in {E_{i}^{s}}\\ t_{u} \in& \{1,...,M\} \: \forall t_{u} \in D_{i}\\ t_{u} &= H_{i-1}(u) \: \forall t_{u} \in C_{i} \end{aligned} $$

Where:

*M* is the maximum number of hierarchy levels

This formulation minimizes the number of conflicting edges, where *ε*_*uv*_ can take either 0 (if (*u*,*v*) is *not* a conflicting edge) or 1 (if (*u*,*v*) is a conflicting edge). The first constraint ensures that if *t*_*u*_≤*t*_*v*_ (which implies (*u*,*v*) is a conflicting edge), then it holds if and only if *ε*_*uv*_ takes 1. While *t*_*u*_>*t*_*v*_ (which implies (*u*,*v*) is *not* a conflicting edge), the condition holds no matter *ε*_*uv*_ takes 0 or 1. But in order to minimize $\sum _{(u,v) \in {E_{i}^{s}}}\epsilon _{\textit {uv}}$, the *ε*_*uv*_ will take 0. The second constraint requires *ε*_*uv*_ to be either 0 or 1. The third constraint assigns nodes in *D*_*i*_ to one of the levels from 1 to *M*. The last constraint says that level assignment for nodes in *C*_*i*_ will be taken from *H*_*i*−1_.

The problem above has $|D_{i}|+|{E_{i}^{s}}|$ variables. Since the topologies of *G*_*i*−1_ and *G*_*i*_ are highly similar (see Definition 6), it is expected that their corresponding assignments *H*_*i*−1_ and *H*_*i*_ also share high similarity. Thus, we expect that the cardinality of the dynamic set *D*_*i*_ to be very small as compared to that of *V*_*i*_. In other words, very small number of nodes are likely to change their hierarchy assignments as graphs evolve gradually. As a result, the number of variables in our ILP formulation is dramatically smaller than the standard ILP solution, such as HIDEN.

##### Dealing with node insertion and deletion

So far we have formulated our D-HIDEN algorithm for solving a sequence of dynamic graphs **G**=(*G*_1_,*G*_2_,…,*G*_*K*_) whose nodes are preserved (i.e., *V*_*i*_=*V*_*j*_,∀*G*_*i*_,*G*_*j*_∈**G**). According to Definition 6 however, the set of nodes may also slightly change as graph evolves. That is, new nodes can be inserted or existing nodes can be removed in a graph in **G** to obtain the next graph in the sequence. We solve this problem as follows. Consider two consecutive graphs *G*_*i*−1_ and *G*_*i*_ in **G** such that *V*_*i*−1_≠*V*_*i*_. To compute the hierarchy of *G*_*i*_, we do not need to define any variable for the nodes that are removed from *V*_*i*−1_ (i.e. the nodes in *V*_*i*−1_−*V*_*i*_). Since we do not know the initial assignment of newly introduced nodes (i.e., nodes in *V*_*i*_−*V*_*i*−1_), we include all of these new nodes in the dynamic node set while creating the ILP constraints.

##### Dealing with disconnected graphs

Another assumption we have previously made is that the graph is connected. For disjointed graphs, we apply D-HIDEN on each of the individual components to obtain hierarchical decompositions. The rationale behind this strategy is the following: as there is no edge between any pair of disjointed components, the decomposition is optimal if and only if the decompositions of these components are optimal.

##### So, what is the challenge?

The ILP formulation we provide above is brief and varies slightly from the traditional ILP solutions for the same problem. So, the obvious question is: What is the challenge here?

The crux of our algorithm lies in creating the dynamic set of nodes *D*_*i*_, which will be detailed in this section. Recall that this set can only be precisely known after the decomposition *H*_*i*_ is actually computed. This information is however not known at this stage. It is actually what is computed at the end of this stage. To deal with this cyclic dependency, we predict the set *D*_*i*_ prior to computing *H*_*i*_. Let us denote our predicted set with ${D^{p}_{i}}$. The performance and the accuracy of our algorithm depends on how well ${D^{p}_{i}}$ represents *D*_*i*_. Thus, the central challenge lies in predicting the dynamic set precisely. In the following, we describe how we address this challenge.

OUR PREDICTION STRATEGY IN A NUTSHELL. In order to predict whether a node will change its hierarchy level as the graph evolves from *G*_*i*−1_ to *G*_*i*_, we learn a probabilistic model that predicts whether a given node will change its hierarchy level based on its neighboring nodes. We do this by learning from a large collection of graphs with random topological perturbations. The rationale behind this local modelling strategy is that the penalty arising from the hierarchy level of a node is determined by its neighboring nodes. This is because the penalty associated with this node involves conflicting edges between itself and its neighbors. We explain our method in detail next.

DETAILED ALGORITHM. Consider two consecutive graphs *G*_*i*−1_ and *G*_*i*_ in a sequence of dynamically evolving graphs, with *H*_*i*−1_ given. We start by defining the following sets which partition the nodes of the evolving graph into three classes based on their movements in the hierarchy.

$$\begin{aligned} \phi_{down}&=\left\{v|H_{i}(v)<H_{i-1}(v)\right\}\\ \phi_{same}&=\left\{v|H_{i}(v)=H_{i-1}(v)\right\}\\ \phi_{up}&=\left\{v|H_{i}(v)>H_{i-1}(v)\right\}\\ \end{aligned} $$

Conceptually, *ϕ*_*down*_, *ϕ*_*same*_ and *ϕ*_*up*_ contain the set of nodes which move down, stay at the same level, or move up in the hierarchy respectively. Next, we define six more sets describing the six possible relationships between neighboring nodes based on their relative positions in the hierarchy.

$$\begin{aligned} \psi_{1}&=\{(u,v)|H_{i-1}(u)>H_{i-1}(v) \wedge (u,v) \in E_{i}\}\\ \psi_{2}&=\{(u,v)|H_{i-1}(u)=H_{i-1}(v) \wedge (u,v) \in E_{i}\}\\ \psi_{3}&=\{(u,v)|H_{i-1}(u)<H_{i-1}(v) \wedge (u,v) \in E_{i}\}\\ \psi_{4}&=\{(u,v)|H_{i-1}(u)>H_{i-1}(v) \wedge (v,u) \in E_{i}\}\\ \psi_{5}&=\{(u,v)|H_{i-1}(u)=H_{i-1}(v) \wedge (v,u) \in E_{i}\}\\ \psi_{6}&=\{(u,v)|H_{i-1}(u)<H_{i-1}(v) \wedge (v,u) \in E_{i}\} \end{aligned} $$

**Figure 4:**
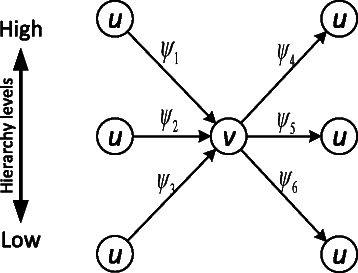
Illustration of the six possible relationships between neighboring nodes ***u*** and ***v*** as denoted with ***ψ***_***1***_ ***,ψ***_***2***_ ***,…,ψ***_***6***_ based on the direction of connecting edges and relative locations of the nodes in the hierarchy.

Figure 4 illustrates the relationships denoted with *ψ*_1_,*ψ*_2_,*ψ*_3_,*ψ*_4_,*ψ*_5_,*ψ*_6_.

Using the sets above, we define two more sets:

$$\begin{aligned} \Phi&=\{\phi_{down},\phi_{same},\phi_{up}\}\\ \Psi&=\{\psi_{1},\psi_{2},\psi_{3},\psi_{4},\psi_{5},\psi_{6}\} \end{aligned} $$

Next, we define a function *f*:*V*×*Φ*→[0,1] such that *f*(*v*,*ϕ*)=*P*(*v*∈*ϕ*) represents the probability that *v*∈*ϕ*. This function tells the probability that for a given node *v*, the probability that this node moves down (*v*∈*ϕ*_*down*_), stays at the same level (*v*∈*ϕ*_*same*_), or moves up (*v*∈*ϕ*_*up*_). Let us denote the set of neighboring nodes of a given node *v* with *N*(*v*)={*u*|(*u*,*v*)∈*E*_*i*_∨(*v*,*u*)∈*E*_*i*_}. In order to compute the function *f*(*v*,*ϕ*), we first need to define two other functions. The first one, denoted with *P*_*ψ*_(*v*∈*ϕ*|*u*∈*ϕ* ^′^), is the probability that *v*∈*ϕ* on the condition that the neighbor node *u*∈*ϕ* ^′^ and (*v*,*u*)∈*ψ* where *ψ*∈*Ψ*. The second one is the prior of the given node *v*, denoted as *f*_0_(*v*,*ϕ*). The prior function *f*_0_ represents the probability of movement in hierarchy for node involved in edge operations as denoted by set *ϕ*. We explain how we compute the function *P*_*ψ*_ and *f*_0_ in detail later in this section. Using these definitions, we formulate *f*(*v*,*ϕ*) as

(1) $$ {\fontsize{8.8pt}{9.6pt}\selectfont{\begin{aligned}{} f(v,\phi)&=\frac{1-\alpha}{|N(v)|}{\sum\nolimits}_{u \in N(v)}{\sum\nolimits}_{\phi' \in \Phi} f\left(u,\phi'\right)P_{\psi} \left(v \in \phi|u \in \phi'\right)\\&\quad +\alpha f_{0}(v,\phi) \end{aligned}}}  $$

The first term in this equation is the normalized average contribution from the neighboring nodes. The second term is the prior of the given node *v*. The parameter *α* is a real number in the [0,1] interval. It controls the relative contributions of these two terms.

Next, we define a vector *x* of size |*V*|×|*Φ*| to describe the relationship of each node in *V* with each class in *Φ*. To simplify our notation, we describe *x* in doubly indexed form as *x*_*v*,*d*(*i*)_=*f*(*v*,*ϕ*_*d*(*i*)_),*v*=1,…,|*V*_*i*_|;*d*=[*d* *o* *w* *n*,*s* *a* *m* *e*,*u* *p*] and *i*=1,…,|*Φ*| We further define a matrix *A* and vector *b* such that

$${} {\fontsize{8.4pt}{9.6pt}\selectfont{\begin{aligned} A((v-1)|\Phi|+i,(v'&-1)|\Phi|+i')=\left\{ \begin{array}{cl} 0 & v' \notin N(v)\\ \frac{P_{\psi}\left(v \in \phi_{d(i)} | v' \in \phi_{d(i')}\right)}{|N(v)|} & otherwise \end{array}\right.\\ &b((v-1)|\Phi|+i)=f_{0}(v,\phi_{d(i)}) \end{aligned}}} $$

Using these definitions, we rewrite Equation (1) as

(2) $$ \begin{aligned} x=(1-\alpha)Ax+\alpha b \end{aligned}  $$

We compute the solution *x* ^∗^ to Equation (2) as

$$\begin{aligned} x^{*}=(I-(1-\alpha)A)^{-1}\alpha b \end{aligned} $$

After solving Equation (2), the probabilities of node *v* to move down, stay the same and move up in the hierarchy are given by $x^{*}_{v,down}$, $x^{*}_{v,same}$ and $x^{*}_{v,up}$, respectively.

Recall that, in order to evaluate Equation (1), we need to compute the prior function *f*_0_ and the function *P*_*ψ*_ that captures the conditional probability of the relative location of a node in the hierarchy conditioned on the hierarchy of its neighboring nodes. In the following, we explain how we compute these two functions in detail.

##### Computation of the prior function

The computation of *f*_0_ involves two parts. The first part trains a model using a large number of random graphs. The second part predicts the value of *f*_0_ for a given graph.

Algorithm 1 presents the first part. Briefly, for training, the algorithm takes two consecutive graphs *G*_1_ and *G*_2_ in the dynamically evolving sequence along with their hierarchical decompositions *H*_1_ and *H*_2_ as input. It then iterates over all the edges (lines 5-27) and identifies the ones that are not common to the two graphs (lines 6-13). These are the edges that have dynamically changed (i.e., inserted or removed) in the input graphs. For all such edges (*u*, *v*), it then counts the number of times we observe that these nodes have moved up, moved down, or stayed at the same level (lines 14-15). Next, it records the same statistics for the neighboring nodes of *u* and *v* (lines 16-25). Finally, it normalizes these counts with the total number of nodes in each class to find the fraction of nodes that move up, move down, or stay at the same level for each class (lines 28-35).

Algorithm 2 presents the second part. This algorithm takes the two consecutive graphs *G*_1_ and *G*_2_ along with the hierarchical decompositions *H*_1_ of *G*_1_, and the model *M* learned in Algorithm 1. It starts by initializing the prior function, so that all the nodes stay at the same level with 100% probability (lines 5-7). This defines the base case, where there is no change in the graph topology. It then iterates over all the edges (lines 9-31) and identifies the ones that are not common to the two graphs (lines 11-17). For each such edge (*u*, *v*), it then updates the prior function for nodes *u*, *v* and all of the neighbors of *u* and *v* using the model *M* (lines 18-29).





**Table 1:** **Algorithms for** MOVEMENT-OF-NODE **and** POSITION-OF-NODE **functions**

MOVEMENT-OF-NODE ** (** ***h***_**1**_ **,** ***h***_**2**_ **)**	POSITION-OF-NODE ** (** ***h***_**1**_ **,** ***h***_**2**_ **)**
	



**Computation of the function** ***P***_***ψ***_ **.** The function *P*_*ψ*_ computes the conditional probability of the relative locations (i.e, are they at the same level or is one above another) of the neighboring nodes in the hierarchy conditioned on each other. Algorithm 3 shows a pseudocode that describes how we learn this function.

The algorithm takes a graph *G*_1_, its hierarchical decomposition *H*_1_, and a potentially large integer *N* denoting the number of random hierarchy perturbations (i.e., number of times, the hierarchy of nodes will be altered) to be used for training as input. It iteratively alters the position of a node *N* times (line 8). At each iteration, it randomly picks a node and moves it to a different level in the hierarchy (lines 9-10). After this alteration, it recomputes the hierarchy of the rest of the nodes by fixing the location of this node at the newly selected location (line 11). We denote this new hierarchy with the function *H*_2_. It then records the movement of the randomly selected node as well as those of all of its neighbors by comparing *H*_1_ and *H*_2_ (lines 12-24). It records the number of times each of the relative movement class is observed over all these neighbors (lines 25-26). Finally, it returns the distribution of the fraction of times each class is observed as the function *P*_*ψ*_.

**Table 2:** **Algorithms for** NEIGHBOR-RELATIONSHIP **and** JOINT-PROBABILITY-NORMALIZATION **functions**

NEIGHBOR-RELATIONSHIP ** (** ***v*** **,** ***w*** **,** ***E*** **,** ***H*** **)**	JOINT-PROBABILITY-NORMALIZATION ** (** ***J*** **)**
	

##### How do we choose the dynamic node set?

So far, we have described how we compute the probability distribution for each node in *G*_*i*−1_ to move up, down, or remain at the same level in the hierarchy based on the topological differences between *G*_*i*−1_ and *G*_*i*_. Recall that the aim of D-HIDEN is to recompute the hierarchy levels for only a small set of nodes (called dynamic node set) that can move in the hierarchy rather than recompute it for the entire graph. The final question we need to answer to complete the description of our method is how we construct the dynamic node set. To find the node in this set, we first sort all the nodes in *G*_*i*_ in ascending order of their probability to stay unchanged in the hierarchy. More specifically, we sort all *v*∈*V*_*i*_ in increasing order of $x^{*}_{v,same}$. The reason behind this ranking strategy is that nodes with higher ranks have higher probability to change their hierarchy levels, and are more preferable to be a part of the dynamic node set.

## Results

In this section, we evaluate the performance of the D-HIDEN method extensively on both synthetic and real datasets. We compare the D-HIDEN method to five currently available methods that can only deal with a static network topology: BFS, Vertex-Sort, HINO, HIDEN, and DC-HIDEN. To the best of our knowledge, our method is the first one to address the hierarchical decomposition problem for dynamically evolving networks. To use them for a sequence of dynamically evolving networks **G**=(*G*_1_,*G*_2_,…,*G*_*K*_), we run each of these competing methods independently on each *G*_*i*_ in **G**. We compute the performance of these methods in terms of the resulting penalty and the running time.

### Datasets

We use both synthetically generated and real gene regulatory network datasets in our experiments.

SYNTHETIC DATA SETS We randomly generate scale-free gene regulatory networks following the Barabasi-Albert model [10] for varying number of nodes and densities (i.e., number of edges per node). Using this model, we create the first network, *G*_1_, in the sequence of dynamically evolving networks for each sequence, **G**=(*G*_1_,*G*_2_,…,*G*_*K*_). Let us denote each network *G*_*i*_ in this sequence with *G*_*i*_=(*V*_*i*_,*E*_*i*_). In order to construct the subsequent networks *G*_*i*_ (1<*i*≤*K*) in each sequence, we mutated the topology of *G*_*i*−1_ using the degree preserving edge shuffling method [25]. This technique is frequently used in the literature to alter network topology while ensuring that all nodes maintain their degrees. Briefly, each mutation step of this technique randomly picks two edges, such that the edges do not share a node. It then swaps the end points of those edges. For instance, assume that the two randomly selected edges are (*u*_1_, *v*_1_) and (*u*_2_, *v*_2_). The mutation step replaces these two edges with (*u*_1_, *v*_2_) and (*u*_2_, *v*_1_). Given a mutation rate *r*≥0, we perform $\left \lceil \frac {r \times |E_{i-1}|}{2}\right \rceil $ mutation steps on *G*_*i*−1_ to generate *G*_*i*_. We use *r*=0.1 as the mutation rate in our experiments (i.e. at most 10*%* of the edges are swapped) unless otherwise specified.

**Figure 5:**
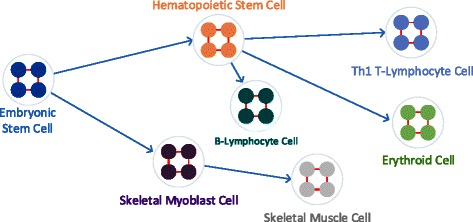
Developmental order among seven human cell types. An edge from cell type *x* to cell type *y* indicates that *x* appears before *y* in development order. Each path from the embryonic stem cell to each of the four cell types Th1 T-Lymphocyte, Erythroid, B-Lymphocyte, and skeletal muscle cell defines a different sequence of dynamically evolving network.

REAL DATA SETS We use the human gene regulatory network dataset of Neph *et al.* 2012 [21]. This dataset contains the regulatory information of the transcription factors for 41 different cell and tissue types. The number of nodes in these networks vary from 493 to 533, and the number of interactions range from 16,461 to 17,320. Among these 41 networks, we select four dynamically evolving sequences, each containing three cell types that are known to follow each other throughout the development stages. The first one contains embryonic stem cells, hematopoietic stem cells and erythroid (K562) cells. The second contains embryonic stem cells, hematopoietic stem cells and Th1 T-Lymphocyte. The third contains embryonic stem cells, hematopoietic stem cells and B-Lymphocyte (CD20+). The last one contains embryonic stem cells, skeletal myoblast and skeletal muscle cells. Figure 5 illustrates these four sequences.

**Quality measures used** We used various quantifiable measures to evaluate the success and the limitations of our method and the competing methods.

- We report the number of conflicting edges (see Definitions 4 and 5) as the penalty of a given hierarchical decomposition. Smaller values of this measure indicates better results.
- Recall that our method aims to accelerate the hierarchical decomposition in dynamically evolving networks by predicting the nodes that are likely to move in the hierarchy. Thus, after the first network in the sequence, errors in prediction may increase the penalty of the resulting decomposition. We report the accuracy of our method in terms of how it compares to the exhaustive method, HIDEN, when HIDEN uses all the nodes and edges as variables for all networks in the sequence. Let us denote the penalties of the methods HIDEN and D-HIDEN with penalty(HIDEN) and penalty(D-HIDEN) respectively. We compute the accuracy as
  $$\frac{1 + \textrm{penalty(HIDEN)}}{1 + \textrm{penalty(D-HIDEN)}}. $$
  Accuracy takes a value in the (0,1] interval. Larger values indicate better results. It is worth mentioning that the mixed integer linear programming solution of the hierarchical decomposition problem can have time complexity exponential in the number of variables used. Therefore, the true optimal result can only be computed for very small networks which are not considered in this paper.

**Implementation and sytem details** We implemented the D-HIDEN, HIDEN, DC-HIDEN, BFS, Vertex-Sort and HINO algorithms using MATLAB. We conducted all the experiments on a hexa core 4.5-GHz CPU (Intel 3960x) 64 GB-memory computer, running the Linux operating system.

### Evaluation of large scale networks

We first compare our method against the state-of-the-art methods in the literature, namely BFS [11], Vertex-Sort [12], HINO [13] and DC-HIDEN [14] for large scale networks. We omit the HIDEN method in this experiment as it does not scale to networks with over 100 nodes.

**Figure 6:**
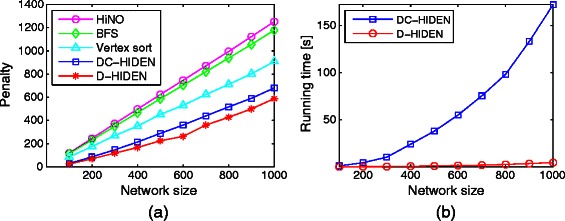
Comparison of the accuracy and running time of D-HIDEN and the state-of-the-art methods on large scale networks.**(a)** Comparison of the penalties arising from the hierarchical decomposition obtained by five methods for different network sizes. **(b)** The running time of D-HIDEN and DC-HIDEN for different network sizes. The results are reported in seconds.

To observe the performance trend as network size varies, we run experiments on different network sizes, ranging from 100 to 1000. For each network size, we randomly create 50 initial networks with edge density 2. Then, we mutate these networks with mutation rate 0.1 to get the corresponding mutated networks in the sequence of dynamically evolving networks. In order to apply our method, we first compute the initial hierarchical decomposition of the first network at each dynamically evolving sequence using the DC-HIDEN method. We then compare the performance of hierarchical decomposition on the mutated networks. Note that for each sequence of networks, the initial decomposition needs to be computed only once. We set the size of dynamic node set for our method (see Definition 7) to 50 for all networks. To ensure that the results are reliable, we repeat this process to create 50 such mutated networks for each network size and report their average penalty and running time (Figure 6).

D-HIDEN yields significantly less penalty than all the competing methods for all network sizes (Figure 6(a)). Thus, it is the most accurate among all competing methods. Furthermore, the gap between the accuracy of D-HIDEN and the rest of the methods grow consistently as network size increases. These results are highly encouraging especially given the fact that D-HIDEN considers updating the hierarchy levels of only a small subset of nodes while all the other methods consider updating all the nodes.

To determine the computational cost of these methods next we compare the running times of the two most accurate methods, D-HIDEN and DC-HIDEN, as the remaining methods have extremely low accuracies. Figure 6(b) shows that D-HIDEN is orders of magnitude faster than DC-HIDEN. As the network size grows, DC-HIDEN’s running time increases exponentially while D-HIDEN’s running time increases linearly. As the network size grows to 1000 nodes, the penalty of the hierarchical decomposition of our method is 15*%* less than DC-HIDEN and it is also about 100 times faster. These results suggest that as the network size increases, our method becomes even more advantageous.

### Evaluation of D-HIDEN under various network characteristics

So far in our experiments, we have demonstrated the superiority of D-HIDEN against other scalable methods. Here, we focus further on our method and compare it against HIDEN. The HIDEN method is exhaustive as it minimizes the penalty while allowing all node and edges to be variables. On the other hand, D-HIDEN creates variables for only a small fraction of the nodes and edges. Therefore, HIDEN yields the smallest possible penalty among all the hierarchical assignments (using the ILP method). Thus, the purpose of the next set of experiments is to determine how close our solutions are to those of HIDEN. We use synthetically generated scale-free networks of different characteristics in these experiments. Due to explosive increase in the running time of the HIDEN method, we limit our experiments to the largest possible network sizes and densities we could run HIDEN on our system.

We focus on three key parameters which affects the performance of hierarchical decomposition in this experiment: number of nodes, network density, and maximum number of hierarchy levels allowed. While evaluating each of these parameters, we fix all the others (Table 3). For each parameter setting, we first randomly generate an initial network. We then mutate that network with mutation rate 0.1 to get subsequent evolving networks. We compute the hierarchy assignment of the first network in each sequence using HIDEN. For the remaining networks in the sequence, we use D-HIDEN to compute the hierarchy incrementally. In order to observe the impact of the size of the dynamic node set, we vary the size of this set from 10*%* to 80*%* of the total number of nodes in the given network. Notice that smaller sizes for this set yield fewer variables in the ILP formulation of D-HIDEN. Inversely, large sizes imply conservative predictions for D-HIDEN allowing for more nodes to alter their hierarchy levels. We also run HIDEN on each network. For each parameter setting, we repeat this experiment 500 times and measure the average penalties and running times.

**Table 3:** **Parameter settings used in our experiments for evaluating the impact of various network characteristics comparison**

**Evaluated**	**Network**	**Number of**	**Number of allowed**
**characteristics**	**density**	**nodes**	**hierarchy levels**
Network density	[1,2,3]	50	5
Number of nodes	2	[30,50,70]	5
Hierarchy levels allowed	2	50	[3,5,10]

Each row corresponds to one experiment where we fix the value of two parameters and vary one.

#### Impact of network density

**Figure 7:**
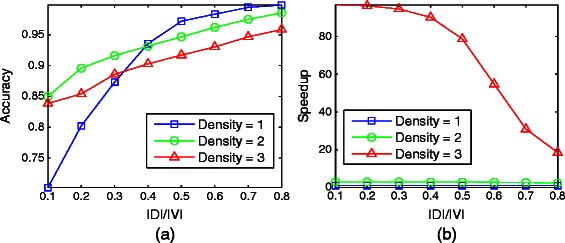
Impact of the network density on D-HIDEN method’s accuracy and running time.**(a)** Accuracy of D-HIDEN for networks with different edge densities computed. **(b)** The speedup of D-HIDEN over HIDEN (i.e., running time of HIDEN divided with the running time of D-HIDEN) for the same experiment. In both figures, *x*-axis represents the ratio of the number of genes in the dynamic node set to that in the entire network.

First, we explore the impact of network density. We fix the number of nodes to 50 and the number of allowable hierarchy levels to 5. We experiment for densities 1,2 and 3. Note that for larger densities, HIDEN did not run till completion due to exponential increase in the running time. For high density networks the accuracy of D-HIDEN is very high even if we use a small fraction of nodes as the dynamic node set (Figure 7(a)). For low density networks, although the accuracy is low for very small dynamic node set, it improves quickly as the size of dynamic node set increases. The running time of our method is significantly faster to that of HIDEN for dense networks (Figure 7 (b)). Especially for small dynamic node sets, the speed up of our method over HIDEN becomes more dramatic. For example at density equal to 3, when the size of the dynamic node set is 40*%* of the node set, our method is almost two orders of magnitude faster than HIDEN. These results are highly encouraging since our method can obtain high accuracies using small dynamic node sets. Collectively these results suggest that our method is highly preferable, especially when the network has medium to high density.

#### Impact of number of nodes

**Figure 8:**
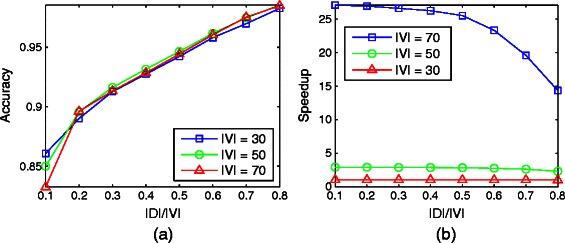
Impact of the number of nodes on D-HIDEN method’s accuracy and running time.**(a)** Accuracy of D-HIDEN for networks with different number of nodes. **(b)** The speedup of D-HIDEN over HIDEN (i.e., running time of HIDEN divided with the running time of D-HIDEN) for the same experiment. In both figures, *x*-axis represents the ratio of the number of genes in the dynamic node set to that in the entire network.

Next, we consider the impact of number of nodes. We fix the density to 2 and the number of allowable hierarchy levels to 5. We experiment for the number of nodes 30,50 and 70. D-HIDEN can achieve a very high accuracy regardless of the network size (Figure 8(a)). For example, when the dynamic node set includes only 20*%* of nodes, D-HIDEN can achieve 90*%* accuracy. This suggests that our method can be applied to large networks without incurring noticeable decrease in accuracy. Figure 8(b) reflects that as the number of nodes increases, the running time of HIDEN increases dramatically while that of D-HIDEN grows gradually. Interestingly, as the size of the dynamic node set increases, the advantage in the running time of D-HIDEN does not noticeably decrease initially (e.g. ratio from 0.1 to 0.5), which allows us to set a higher ratio for D-HIDEN to achieve better accuracy without too much additional cost in running time. In summary, these results suggest that our method is favorable, especially when the size of networks gets larger.

#### Impact of the number of allowed hierarchy levels

**Figure 9:**
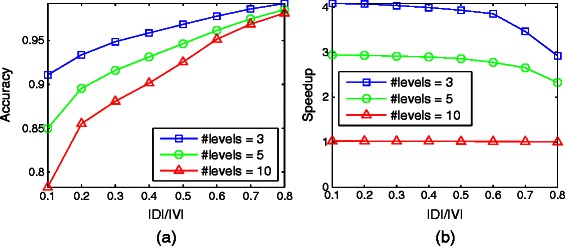
Impact of the number of allowed hierarchy levels on D-HIDEN method’s accuracy and running time.**(a)** Accuracy of D-HIDEN for networks with different numbers of allowed hierarchy levels. **(b)** The speedup of D-HIDEN over HIDEN (i.e., running time of HIDEN divided with the running time of D-HIDEN) for the same experiment. In both figures, *x*-axis represents the ratio of the number of genes in the dynamic node set to that in the entire network.

Finally, we focus on the impact of maximum hierarchy level. We fix the density to 2 and the number of nodes to 50. We experiment for the number of allowed hierarchy levels 3,5 and 10. We observe a very high accuracy of D-HIDEN (Figure 9(a)). As the number of allowed hierarchy levels decreases, the accuracy increases consistently, because the number of nodes that need to change their hierarchy levels also decreases. Based on these results, we can conclude that D-HIDEN consistently achieves extremely high accuracy, especially for small number of allowed hierarchy levels. Figure 9(b) suggests that the running time is relatively insensitive to the number of allowed hierarchy levels.

### Evaluation of the impact of the number of dynamic steps

So far, we have analyzed the performance of our method for only one dynamic step. In other words, in the sequence of dynamically evolving networks **G**=(*G*_1_,…,*G*_*K*_), *K* was set to 2. In this experiment, we evaluate how the performance of our method is affected by the length of this sequence (i.e. *K*). The purpose is to explore how the error propagates as the deviation between the topology of the subsequent networks from that of the initial network grows with increasing value of *K*.

#### Evaluation on large networks

In this experiment, we generate synthetic network with 500 nodes and density equal to 2 to use as the initial network *G*_1_ in the sequence. We then create 25 subsequent networks *G*_1_,*G*_2_,…,*G*_25_. To construct each *G*_*i*_ (1<*i*≤25), we mutate *G*_*i*−1_ with 0.1 mutation rate. We repeat this process 1000 times to construct 1000 such independent sequences of dynamically evolving networks. Because these networks are too large for the HIDEN method, we compare our method to the most accurate competing method (DC-HIDEN) which can solve networks of this size.

**Figure 10:**
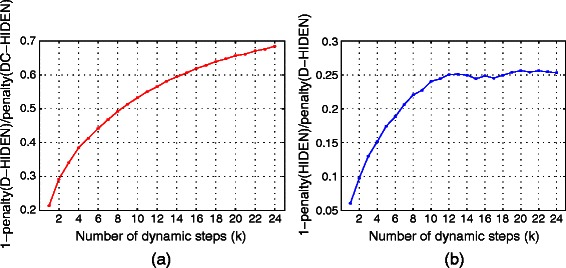
Performance comparison of D-HIDEN to DC-HIDEN methods when the regulatory network is continuously modified.**(a)** Relative penalty performance of DC-HIDEN and D-HIDEN on large networks. **(b)** Relative penalty performance of D-HIDEN and HIDEN on small networks. In both figures, *x*-axis represents the number of mutation steps. At each mutation step, the topology of the previous network is altered by 0.1 mutation rate.

We observe that as the number of mutation step increases, the advantage of our method over the DC-HIDEN enlarges (Figure 10(a)). Initially at mutation step 1, our method has about 20*%* less penalty than the DC-HIDEN, and when the mutation step reaches 24, our method has 68*%* less penalty than the DC-HIDEN. This reveals that the accuracy of our method relative to that of DC-HIDEN gets better on continuously changing networks with growing number of networks in the sequence. Though the initial hierarchy assignments of base networks were inaccurate (as they were computed with DC-HIDEN), at each mutation step our method has an opportunity to identify the nodes whose hierarchy levels should be updated, and sets them as variables instead of constants. Thus, it corrects the hierarchy of new set of nodes each dynamic step.

#### Evaluation on small networks

Next, we use networks with a small number of nodes to compare the performance of our method against the exhaustive HIDEN method. More specifically, we construct networks with 50 nodes and density equal to 2. Similar to the previous experiment, we generate sequences of dynamically evolving networks with 25 networks in each sequence, and use 0.1 mutation rate while altering the topology of consecutive networks. We repeat this process 1000 times and report the average results.

We observe that as the number of mutation steps increase, the advantage of HIDEN over our method enlarges (Figure 10 (b)). This reveals that HIDEN is intrinsically better than our method in terms of accuracy only (note that again the computational cost of HIDEN is exponentially higher than our method as it exhausitively considers all nodes in all networks. Thus, for networks with over 100 nodes, it becomes intractable). The reason behind this trend is also similar to the previous experiment but this time the initial assignment of base networks were computed by HIDEN. Interestingly the curve converges quickly around step 10, where the penalty of our method is only about 25*%* higher than HIDEN. This is very promising, because even after mutating the initial network 24 times with 10*%* mutation at each step, the error incurred by our method does not grow significantly. This suggests that our method does not diverge from the optimal solution greatly even for a large number of dynamic steps.

### Results on real data

To assess the applicability of our method to real biological regulatory networks, in this section we analyze four dynamically evolving cell lineages each containing three cell types. In the first three cell lineages embryonic stem cells are developed into hematopoietic stem cells, and the hematopoietic stem cells are developed into erythroid, T-lymphocyte and B-lymphocyte respectively. In the fourth cell lineage, embryonic stem cells develop into skeletal myoblast and eventually to skeletal muscle.

#### Penalty comparison on real data

In the above sections, we have demonstrated the superiority of D-HIDEN against the state of the art methods BFS, Vertex-Sort, HINO and DC-HIDEN on synthetic datasets. To show the applicability of our method to real gene regulatory networks, here we compare the accuracy of the D-HIDEN method to these four methods on a real dataset (described above). In this comparison, we focus on the gene regulatory networks of the seven cell types in our dataset: hESC, CD34+, HSMM, K562, Th1, CD20+ and SKMC. To apply our method; we first find the initial hierarchical decomposition of the hESC gene regulatory network (first network in the chain) using DC-HIDEN method and then determine the hierarchy assignments of the genes in the rest of the cells using D-HIDEN. In the final step, we compute the penalties for each cell-type based on the resulting hierarchy assignments. We apply the other methods as follows; we first find the hierarchical decomposition of all the networks in different cell types using the specific method and then compute the penalties for each network based on the resulting assignments.

**Figure 11:**
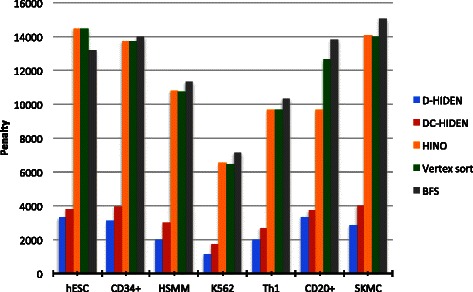
Comparison of the penalties arising from the hierarchical decomposition obtained by five methods on real datasets. In the figure, *x*-axis represents the seven cell types and *y*-axis represents the penalty score.

Our results demonstrate that D-HIDEN yields significantly less penalty than all the competing methods (Figure 11). Thus it is the most accurate among the competing methods on gene regulatory networks of the seven cell types. We observe that the penalty score of the HINO, Vertex Sort and BFS methods are usually at least 3 fold more than the D-HIDEN method. Similarly, D-HIDEN method is more accurate than the DC-HIDEN method. The superior accuracy of D-HIDEN method on real datasets strengthens our analysis on the synthetic datasets, and suggests that our method can be used to create highly accurate hierarchical decomposition of real datasets.

#### Biological derivations on real data

Here, we apply our method to four dynamically evolving cell linages described above. The experimental results reveal the changing hierarchy of gene regulation in these four lineages of embryonic stem cells (Tables 4 and 5). Similar trends can be observed for each cell lineage. In the lineage of embryonic stem cells (H7-hESC) that develop into erythroid cells (K562), most genes (146 genes) are expressed at level five the highest level in the genetic hierarchy at the embryonic stem cell stage (Table 4). At the erythroid stage, most genes (156 genes) are at level one, which is the lowest level in the hierarchy. In addition, the highest number of genes (533 genes) is observed at the embryonic stem cell stage and this number gradually decreases in each of the following two developmental stages. These two observations are also valid for the cell lineages that develop into T-lymphocyte (Th1) and B-lymphocyte (CD20+) cells. In the cell lineage that develop into skeletal muscle cells (SKMC) the former trend is also observed but the latter is not, in that the number of genes observed at the final stage, skeletal muscle cell, is greater than the number observed in the intermediate, skeletal myoblast (HSMM) stage. It is important to emphasize that as these stem cells develop, more genes move down in the hierarchy (Table 5) in comparison to moving up, which suggests that in general the role of genes as key regulators become less important since they regulate fewer downstream target genes. We observe that most of the genes do not change their hierarchy level, which suggests that the network rewiring is substantial, but it does not involve most of the genes. It is also interesting to note that genes do not fluctuate in their rankings very often. The changes in the hierarchy levels of all of these genes should be analyzed in detail to understand their changing roles in different cell lines.

**Table 4:** **Distribution of the number of genes to different levels for each cell line**

**Level**	**hESC**	**CD34+**	**HSMM**	**K562**	**Th1**	**CD20+**	**SKMC**
1	106	117	122	156	152	157	170
2	95	94	91	90	95	92	90
3	94	90	91	73	74	83	80
4	92	90	89	71	81	73	75
5	146	135	130	103	116	110	114
Total	533	526	523	493	518	515	529

**Table 5:** **Number of genes that move up, move down, stay same, or fluctuate in hierarchy rankings in different cell lineages during the cell development**

**Cell type**	**Move up**	**Move down**	**Stay same**	**Fluctuate**
K562	52	105	317	18
Th1	64	104	332	8
CN20+	53	118	328	8
SKMC	44	119	337	16

For example the MYOG gene moves up in the hierarchy for skeletal muscle cell lineage. Myogenin (MYOG) is the second muscle-specific gene to be expressed in embryonic stem cells in the process of muscle cell development [26]. In a study in myoblasts Brunetti and Goldfine found MYOG to be important in muscle cell development [27]. Mice with a mutation in MYOG could develop myoblasts but the myoblasts could not further form functional myofibers; i.e. MYOG is important for later stages of muscle cell differentiation [28]. In addition, Hasty *et al.* 1993 found that when MYOG is knocked out in mice embryos, the embryos develop but die after birth [29]. These results are consistent with this gene moving up in the genetic hierarchy, as it becomes important later in the muscle cell development. One gene that moves down in the muscle cell lineage is MEF2C because of its importance in early myoblast development. In embryonic stem cells, this gene is used as a marker of cardiac cells and its expression is found to be increased with greater expression of miR-499 [30]. In myoblasts, this gene is an important indicator of early muscle cell differentiation activity because of its importance in regulating skeletal muscle differentiation [31,32]. Taken together, genes that move up or down in the hierarchy seem to have a change in their function and relative importance. The literature supports this conclusion, although more research should be performed to assess the exact functions of these genes in these specific cell types at different stages in cell development.

STAT5 is another gene that moves up the hierarchy in the erythroid cell lineage. When active STAT5 is expressed in embryonic stem cells, the cells are more likely to undergo formation of hematopoietic stem cells (CD34+) [33]. STAT5 is suggested to be important for cell proliferation in hematopoietic stem cells. Overexpression of STAT5 in hematopoietic stem cells resulted in greater cell growth of these cells through the PI3-kinase/AKT pathway [34]. Furthermore, if STAT5 is over expressed in hematopoietic stem cells (CD34+) there is increased renewal of these cells and the hematopoietic stem cells are much more likely to acquire erythroid cell fate [35]. Other researchers have found that in erythroid cells STAT5 is in its active, phosphorylated state and when STAT5 expression is knocked out, these cells exhibit reduced growth in vitro [36]. One gene that moves down in the erythroid cell lineage is GFI1. In embryonic stem cells, GFI1 and GFI1B are downstream targets of RUNX signaling and when RUNX is knocked out, GFI1 along with GFI1B promote hematopoiesis as in the studies with RUNX-/-, the cells display increased expression of the hematopoietic cell marker CD41 as well as a rounded shape [37]. This study suggests that in normal embryonic stem cell development, RUNX expression causes GFI1 expression to be reduced. Since GFI1 may prevent proliferation of hematopoietic stem cells [38], and GFI1 is a downstream target of C/EBP *α*, which prevents cell proliferation when GFI1 levels are low not all cells will become hematopoietic [39]. Thus the roles for the STAT5 and GFI1 genes are changing through development and it is likely that the hierarchy levels of these genes are consequently changing in the gene regulatory network. Our analysis suggests that the STAT5 gene moves up in hierarchy, and GFI1 moves down in the hierarchy. The literature cited above is not clear about the relative importance of these genes in different cell types. So, our method’s predictions suggest potentially new experiments to elucidate the changing role of these genes in different cell types. Clearly, a thorough experimental study is needed to find the relative position of these genes in the regulatory hierarchy in different cell types.

## Discussion and conclusion

Biological systems are tightly controlled, and more importantly, they are affected by dynamic changes in the gene regulatory networks. Because of that, understanding the dynamic changes in gene regulatory networks is critical for a true comprehension of biological systems. One particularly important organizational feature of the gene regulatory networks is the hierarchy of the interactions. Hierarchy in gene regulatory networks describes the flow of control in biological systems. Major experimental and computational efforts are performed to establish the gene regulatory networks; however finding hierarchy in these networks is very challenging. There have been studies to discover the hierarchy in gene regulatory networks, however these studies mainly focused on static networks.

In this study, we presented a novel method named D-HIDEN for discovering hierarchy in dynamic gene regulatory networks. To the best of our knowledge, this is the first method which can incrementally update the hierarchy in a sequence of dynamically evolving networks. D-HIDEN formulates the hierarchy level assignment problem as a local mixed integer linear programming problem. We compared the D-HIDEN method to five currently available methods, namely BFS, Vertex-Sort, HINO, HIDEN and DC-HIDEN, on synthetic and real gene regulatory networks. Our analysis on these networks demonstrated that the D-HIDEN method outperforms the other five methods in terms of minimizing the conflicting edges in hierarchy. In addition to the superior accuracy level of D-HIDEN methods, the running time for this method was also faster than the DC-HIDEN method, which is the next best method in terms of accuracy. Application of the D-HIDEN method to the human gene regulatory networks shows that human genes’ hierarchy levels change dynamically. These changes in the hierarchy levels are not random and reflect functional changes in the network. This method could be applied to other biological datasets such as in cancer biology or neuronal development.

## References

[1] She M, Ye X, Yan Y, Howit C, Belgard M, Ma W (2011). Gene networks in the synthesis and deposition of protein polymers during grain development of wheat. Funct Integr Genomics.

[2] Watson E, Walhout AJM. Caenorhabditis elegans metabolic gene regulatory networks govern the cellular economy. Trends Endocrinol Metab. 2014. doi:10.1016/j.tem.2014.03.004. 10.1016/j.tem.2014.03.004 PMC4178166 24731597

[3] Peter IS, Davidson EH (2011). Evolution of gene regulatory networks controlling body plan development. Cell.

[4] Ó’Maoiléidigh DS, Graciet E, Wellmer F (2014). Gene networks controlling Arabidopsis thaliana flower development. New Phytol.

[5] Buckingham M, Rigby PWJ (2014). Gene regulatory networks and transcriptional mechanisms that control myogenesis. Dev Cell.

[6] Lander AD (2013). How cells know where they are. Science.

[7] Csikász-Nagy A, Palmisano A, Zámborszky J (2011). Molecular network dynamics of cell cycle control: transitions to start and finish. Methods Mol Biol.

[8] Belz GT, Nutt SL (2012). Transcriptional programming of the dendritic cell network. Nat Rev Immunol.

[9] Barabási A-L, Oltvai ZN (2004). Network biology: understanding the cell’s functional organization. Nat Rev Genet.

[10] Barabási A AR (1999). Emergence of Scaling in Random Networks. Science.

[11] Yu H, Gerstein M (2006). Genomic analysis of the hierarchical structure of regulatory networks. Proc Natl Acad Sci USA.

[12] Jothi R, Balaji S, Wuster A, Grochow JA, Gsponer J, Przytycka TM (2009). Genomic analysis reveals a tight link between transcription factor dynamics and regulatory network architecture. Mol Syst Biol.

[13] Hartsperger ML, Strache R, Stümpflen V. HiNO: An approach for inferring hierarchical organization from regulatory networks. PLoS ONE. 2010; 5. doi:10.1371/journal.pone.0013698. 10.1371/journal.pone.0013698 PMC2973965 21079808

[14] Gulsoy G, Bandhyopadhyay N, Kahveci T. HIDEN: Hierarchical decomposition of regulatory networks. 2012. doi:10.1186/1471-2105- 13-250. 10.1186/1471-2105-13-250 PMC3556311 23016513

[15] Bhardwaj N, Kim PM, Gerstein MB (2010). Rewiring of transcriptional regulatory networks: hierarchy, rather than connectivity, better reflects the importance of regulators. Sci Signaling.

[16] Bhardwaj N, Yan K-K, Gerstein MB (2010). Analysis of diverse regulatory networks in a hierarchical context shows consistent tendencies for collaboration in the middle levels. Proc Nat Acad Sci USA.

[17] Ma H-W, Buer J, Zeng A-P (2004). Hierarchical structure and modules in the Escherichia coli transcriptional regulatory network revealed by a new top-down approach. BMC Bioinformatics.

[18] Ma H-W, Kumar B, Ditges U, Gunzer F, Buer J, Zeng A-P (2004). An extended transcriptional regulatory network of Escherichia coli and analysis of its hierarchical structure and network motifs. Nucleic Acids Res.

[19] Cosentino Lagomarsino M, Jona P, Bassetti B, Isambert H (2007). Hierarchy and feedback in the evolution of the Escherichia coli transcription network. Proc Nat Acad Sci USA.

[20] Freyre-González JA, Alonso-Pavón JA, Treviño-Quintanilla LG, Collado-Vides J (2008). Functional architecture of Escherichia coli: new insights provided by a natural decomposition approach. Genome Biol.

[21] Neph S, Stergachis AB, Reynolds A, Sandstrom R, Borenstein E, Stamatoyannopoulos JA (2012). Circuitry and dynamics of human transcription factor regulatory networks. Cell.

[22] Han J-DJ, Bertin N, Hao T, Goldberg DS, Berriz GF, Zhang LV (2004). Evidence for dynamically organized modularity in the yeast protein-protein interaction network. Nature.

[23] Babu MM, Luscombe NM, Aravind L, Gerstein M, Teichmann SA. Structure and evolution of transcriptional regulatory networks. 2004. doi:10.1016/j.sbi.2004.05.004. 10.1016/j.sbi.2004.05.004 15193307

[24] Harbison CT, Gordon DB, Lee TI, Rinaldi NJ, Macisaac KD, Danford TW (2004). Transcriptional regulatory code of a eukaryotic genome. Nature.

[25] Scott J, Ideker T, Karp RM, Sharan R (2006). Efficient Algorithms for Detecting Signaling Pathways in Protein Interaction Networks. J Comput Biol.

[26] Rohwedel J, Maltsev V, Bober E, Arnold HH, Hescheler J, Wobus AM (1994). Muscle cell differentiation of embryonic stem cells reflects myogenesis in vivo: developmentally regulated expression of myogenic determination genes and functional expression of ionic currents. Dev Biol.

[27] Brunetti A, Goldfine ID (1990). Role of myogenin is myoblast differentiation and its regulation by fibroblast growth factor. J Biol Chem.

[28] Arnold HH, Braun T (1996). Targeted inactivation of myogenic factor genes reveals their role during mouse myogenesis: A review. Int J Dev Biol.

[29] Hasty P, Bradley A, Morris JH, Edmondson DG, Venuti JM, Olson EN (1993). Muscle deficiency and neonatal death in mice with a targeted mutation in the myogenin gene. Nature.

[30] Wilson KD, Hu S, Venkatasubrahmanyam S, Fu J-D, Sun N, Abilez OJ (2010). Dynamic microRNA expression programs during cardiac differentiation of human embryonic stem cells: role for miR-499. Circ Cardiovasc Genet.

[31] Gossett LA, Kelvin DJ, Sternberg EA, Olson EN (1989). A new myocyte-specific enhancer-binding factor that recognizes a conserved element associated with multiple muscle-specific genes. Mol Cell Biol.

[32] Potthoff MJ, Olson EN (2007). MEF2: a central regulator of diverse developmental programs. Dev (Cambridge, England).

[33] Kyba M, Perlingeiro RCR, Hoover RR, Lu C-W, Pierce J, Daley GQ (2003). Enhanced hematopoietic differentiation of embryonic stem cells conditionally expressing Stat5. Proc Nat Acad Sci USA.

[34] Santos SC, Lacronique V, Bouchaert I, Monni R, Bernard O, Gisselbrecht S (2001). Constitutively active STAT5 variants induce growth and survival of hematopoietic cells through a PI 3-kinase/Akt dependent pathway. Oncogene.

[35] Schuringa JJ, Chung KY, Morrone G, Moore MAS (2004). Constitutive activation of STAT5A promotes human hematopoietic stem cell self-renewal and erythroid differentiation. J Exp Med.

[36] de Groot RP, Raaijmakers JA, Lammers JW, Jove R, Koenderman L (1999). STAT5 activation by BCR-Abl contributes to transformation of K562 leukemia cells. Blood.

[37] Lancrin C, Mazan M, Stefanska M, Patel R, Lichtinger M, Costa G (2012). GFI1 and GFI1B control the loss of endothelial identity of hemogenic endothelium during hematopoietic commitment. Blood.

[38] Hock H, Hamblen MJ, Rooke HM, Schindler JW, Saleque S, Fujiwara Y (2004). Gfi-1 restricts proliferation and preserves functional integrity of haematopoietic stem cells. Nature.

[39] Lidonnici MR, Audia A, Soliera AR, Prisco M, Ferrari-Amorotti G, Waldron T (2010). Expression of the transcriptional repressor Gfi-1 is regulated by C/EBP *α* and is involved in its proliferation and colony formation-inhibitory effects in p210BCR/ABL-expressing cells. Cancer Res.

